# Exploration of upstream and downstream mechanisms of the TAGLN2 gene in pulmonary arterial hypertension

**DOI:** 10.1097/MD.0000000000045295

**Published:** 2025-10-17

**Authors:** Yu-Shuo Pan, Guo-Rui Xu, Ni-Ni Qu, Yi-Bing Qin, Tie-Fa Guan, Ming-Zhuo Cao, Hong-Wei Bai

**Affiliations:** a Liaoning University of Traditional Chinese Medicine, Shenyang, Liaoning Province, China; b The First Affiliated Hospital of Liaoning University of Traditional Chinese Medicine, Shenyang, Liaoning Province, China; c The Second Affiliated Hospital of Liaoning University of Traditional Chinese Medicine, Shenyang, Liaoning Province, China.

**Keywords:** acetylation, DNA methylation, immune cells, Mendelian randomization, metabolism, PAH

## Abstract

This study systematically explored the upstream and downstream mechanisms of the TAGLN2 gene regulated by DNA methylation and its succinylation modification in the pathogenesis of pulmonary arterial hypertension (PAH) by integrating Mendelian randomization and mediation analysis. The results showed that DNA methylation sites cg13892570 and cg16107628 significantly downregulated TAGLN2 expression (β = −0.53 and −0.61, *P* < .05), indirectly reducing the risk of PAH. The mediating effects of TAGLN2 accounted for 86.46% and 97.65% of the total effect, respectively. Further analysis indicated that TAGLN2 might promote PAH progression through immune and metabolic pathways: on the one hand, by activating HLA DR^+^ monocytes (mediation proportion 6.15%), it exacerbated pulmonary vascular inflammatory responses; on the other hand, by increasing the levels of eicosadienoic acid (mediation proportion 5.21%) and cysteinyl dipeptide (mediation proportion 3.15%), it induced oxidative stress and lipid metabolism disorders. Bioinformatics validation suggested that TAGLN2 was significantly overexpressed in the lung tissues of PAH patients (*P* < .05), and its succinylation modification might enhance the pro-PAH effect by altering protein stability and function. This study first proposed the “DNA methylation-TAGLN2 succinylation-immune/metabolic” regulatory axis, providing new ideas and potential targets for the epigenetic mechanism and precise treatment of PAH. However, the relevant conclusions still need to be further verified by subsequent experiments.

## 1. Introduction

Pulmonary arterial hypertension (PAH) is a fatal disorder characterized by progressive pulmonary vascular remodeling, elevated pulmonary artery pressure, and right heart failure, with a 5-year survival rate of only 60% to 70%. Despite recent advancements in prognosis for some patients through targeted therapies (e.g., endothelin receptor antagonists, PDE5 inhibitors), the pathogenesis of PAH remains insufficiently understood, and early diagnosis and precise treatment strategies are lacking.^[1]^ Accumulating evidence suggests that PAH is not only linked to vascular contractility abnormalities but also strongly associated with metabolic reprogramming, epigenetic regulation, and posttranslational modifications (PTMs) of proteins.^[2–4]^

Recently, lysine succinylation, an emerging PTM, has drawn significant attention due to its critical role in metabolic regulation and cellular signaling. As a novel acyl modification involving carboxylic acids, lysine succinylation is a naturally occurring modification.^[5]^ This modification is implicated in virtually all biological processes, influencing various metabolic pathways, enzyme activity, gene expression, and key metabolic hubs such as the tricarboxylic acid cycle. Its widespread impact highlights its role in regulating energy production, amino acid metabolism, fatty acid synthesis, and redox balance.^[6]^ Moreover, succinylation has been found to be closely associated with the onset and progression of cardiovascular diseases, cancer, neuroinflammatory disorders, metabolic diseases, and liver, lung, and age-related conditions.^[7]^ However, the specific role of succinylation in PAH remains underexplored.

PAH is a multifactorial vascular disorder, with its pathogenesis involving disruptions in intricate molecular regulatory networks. Recent studies have highlighted the significant role of the interplay between epigenetic regulation and metabolic reprogramming in the onset and progression of PAH. DNA methylation, a highly stable epigenetic modification, has been found to exhibit aberrant patterns across the genome in patients with PAH, potentially influencing disease progression by modulating the expression of key genes.^[8]^ Additionally, succinylation, a recently recognized PTM of metabolism-related proteins, maintains a dynamic balance intimately linked to cellular metabolic status, which is notably disrupted in PAH. Despite these insights, 3 critical questions remain unresolved: first, whether DNA methylation contributes to PAH pathogenesis by regulating specific succinylation-related genes; second, whether this regulation further impacts downstream metabolite levels; and third, how these molecular alterations facilitate PAH progression through immune microenvironment remodeling. Addressing these questions is essential for elucidating the novel mechanisms underlying the epigenetic-metabolic-immune regulatory network in PAH.

This study aims to investigate the regulatory role of DNA methylation on specific succinylation-related genes and their downstream effects. The first objective is to examine the causal relationship between succinylation-related genes and PAH using 2-sample Mendelian randomization (MR), a robust approach for inferring causality between genetic variants and complex traits, minimizing confounding biases typical of observational studies.^[9,10]^ Utilize genome-wide association studies (GWAS) and expression quantitative trait loci data to enhance statistical power and identify pathogenic genes.^[11]^ The second objective is to use mediation analysis to explore the mechanistic relationships between DNA methylation and succinylation genes, as well as between succinylation genes and immune cells and metabolites in PAH, decomposing the total effect into direct and indirect components. These investigations are anticipated to provide critical theoretical insights for early diagnosis and precision treatment of PAH and offer novel perspectives on the application of epigenetics in pulmonary vascular diseases.

## 2. Method

### 2.1. Data source

PAH GWAS data

- Database: Finnish FinnGen database (https://www.finngen.fi/en).^[12]^
- Dataset: finnen_r12\u i9\u hyptensul.gz
- Sample size: 301, 0.87% patients with PAH vs 345,634, 99.91% controls.
- Demographic characteristics: female 65.8%, male 34.2%, median age 61.27.
- Diagnostic criteria: International Classification of Diseases-8/9/10 disease classification coding.
- Quality control: data were verified using medical records and clinical diagnoses, with hypertension cases excluded.
- Details: https://risteys.finngen.fi/endpoints/I9_HYPTENSPUL
- Statistical efficacy: the adequacy of the sample size was verified using the MRPower online tool (https://shiny.cnsgenomics.com/mRnd/).

Succinylation gene data

- Source literature: research by Zhao et al,^[13]^ Cheng et al,^[14]^ Hou et al,^[7]^ Shen et al,^[15]^ and Kubatzky et al.^[16]^
- Number of genes: 19 in total (including ACOX1, SIRT5, SOD1, TAGLN2, etc).
- Expression quantitative trait loci data: from eqtlgen database (https://eqtlgen.org) covering 10 succinylation genes.
- Details: see Table S1, Supplemental Digital Content, https://links.lww.com/MD/Q396.

DNA methylation data

- Database: GoDMC database.
- Characteristics: genome-wide scan meta-analysis results based on 420,509 methylation sites.
- Access links: http://mqtldb.godmc.org.uk/downloads.
- Supplementary data: information on the methylation sites of succinylated genes can be downloaded from https://ngdc.cncb.ac.cn/ewas.

Metabolomic and immunogenic data

- Database: GWAS catalog.
- Data volume: 731 types of immune cells; 1400 types of metabolites.

• ID range: The metabolite data IDs range from GCST90199621 to GCST90201020,^[17]^ while the immune cell data IDs range from GCST90001391 to GCST90002121.^[18]^

- Access links: https://www.ebi.ac.uk/gwas/.

### 2.2. MR analysis of the relationship between succinylation and PAH

In this study, “TwoSampleMR” package, “ggplot2” (version 3.5.0) package, and “foreach” (version 1.5.2) package in R language (version 4.3.2) were applied to conduct MR analysis to estimate the potential causal relationship between genetic-predicted succinylation genes and PAH.

Single nucleotide polymorphism (SNP) screening criteria:

- Significant correlation with gene expression (*P* < 5 × 10^−8^).
- *F*-statistic > 10 (to avoid weak instrument bias).
- Removal of linkage disequilibrium (window size 10,000 kb, *R*^2^ < 0.001).

Selection of statistical methods: for succinylation genes with a single SNP, the Wald ratio method was employed; for genes with 2 or more SNPs, causal relationships were estimated using inverse variance weighting (IVW), MR-Egger, weighted median, simple mode, and weighted mode methods, with IVW serving as the primary analytical approach. IVW results were considered the principal basis for drawing conclusions.

### 2.3. Sensitivity analysis

To ensure the robustness of the research findings, multidimensional sensitivity analyses were conducted:

Genetic variation heterogeneity test:

- Cochran *Q* test.
- *P* > .05 indicates no heterogeneity among instrumental variables.

Level pleiotropy assessment:

- Intercept term of MR-Egger regression analysis.
- *P* > .05 indicates the absence of directional pleiotropy.

Outlier detection and correction:

- Application of MR-PRESSO method.
- Global test *P* > .05 confirming no pleiotropic outliers.
- Identification and elimination of potential abnormal SNPs.

Leave-one-out sensitivity analysis:

- Stepwise exclusion of individual tool variables.
- Repeated MR analysis to assess stability.
- Ensuring results are not overly affected by specific SNPs.

### 2.4. Bioinformatics analysis and verification of expression levels

To strengthen the evidence chain and systematically delineate the complete regulatory axis from genetic variation to gene expression and pathological phenotype, the PAH dataset from the GEO database (https://www.ncbi.nlm.nih.gov/geo/) was employed for gene expression validation. The search strategy combined the keywords “Pulmonary Arterial Hypertension” and “RNA-seq,” with a sample size criterion of ≥10, including transcriptome data from both patients with PAH and normal lung tissues. The R package “GEOquery” (version 3.18) facilitated direct download and parsing of the data, while “limma” (version 3.50.0) was used for differential expression analysis. Expression values of succinylated genes were extracted using “ggplot2” to generate box plots. The standardized methods and statistical thresholds used are presented in Table 1.

**Table 1:** The standardized methods and statistical values used in the validation of the GEO dataset.

Step	Method/function	Purpose	Key output/concept
Preprocessing	impute.knn()	Impute missing values	Complete data matrix
	avereps()	Consolidate duplicate genes	Unique gene list
	normalizeBetweenArrays()	Remove technical variation between samples	Expression matrix with consistent distributions
Model fitting	lmFit()	Fit a linear model	Baseline expression levels for each group
	makeContrasts(), contrasts.fit()	Define and compute inter-group differences	logFC (Effect size)
Statistical inference	eBayes()	Stabilize variance estimates	Moderated *t*-statistic
	topTable()	Perform hypothesis testing	P.Value; adj.P.Val (FDR)
Filtering	abs(logFC) ≥ 1	Identify significantly differentially expressed genes	List of DEGs
	adj.P.Val < 0.05		

DEGs = differentially expressed genes.

### 2.5. Mediating analysis of the relationship between DNA methylation, succinylation, and PAH

**Figure 1.**
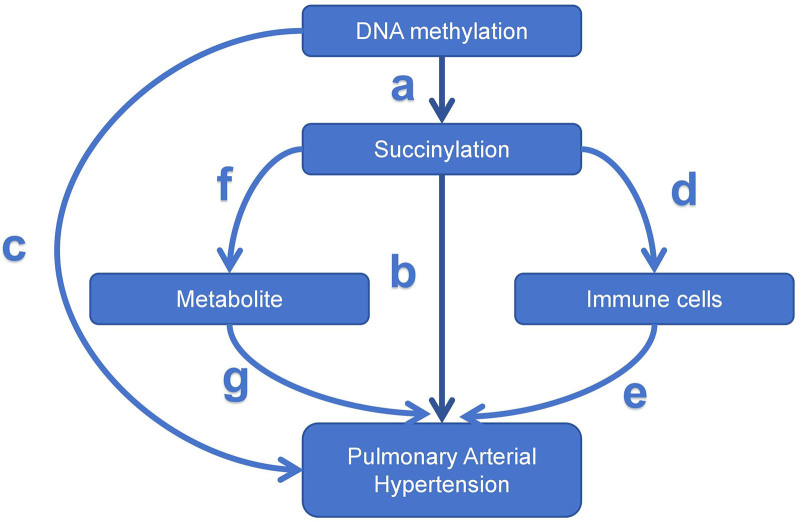
Mediation analysis model illustrating the relationships between DNA methylation, succinylation genes, immune cells, metabolites, and PAH risk. Pathway a: Effect of DNA methylation sites on succinylation gene expression. Pathway b: Effect of succinylation gene expression on PAH risk. Pathway c: Direct impact of DNA methylation on PAH risk. Pathway d: Effect of succinylation genes on immune cells. Pathway e: Effect of immune cells on PAH. Pathway f: Effect of succinylation genes on metabolites. Pathway g: Effect of metabolites on PAH. PAH = pulmonary arterial hypertension.

To investigate the relationship between DNA methylation and succinylation, as well as the role of succinylation in mediating the link between DNA methylation and PAH, a 2-sample MR and mediation analysis were performed (Fig. 1). This approach decomposes the total effect into both direct (unmediated) and indirect (mediated by the mediator) components. Specifically, the overall impact of DNA methylation on PAH consists of 2 parts:

- The direct effect of DNA methylation on PAH (pathway c in Fig. 1).
- The indirect effect mediated by succinylation (pathway a × b in Fig. 1).

The ratio of the indirect effect to the total effect quantifies the potential mediating role of succinylation in this relationship. Given that mediation analysis assumes the absence of unmeasured confounding, genetic instruments were employed to minimize confounding, and sensitivity analyses were conducted to assess the robustness of the results.

The “GEOquery,” “minfi” (version 1.54.1), and “IlluminaHumanMethylation450kanno.ilmn12.hg19” (version 0.6.1) packages in R language were used to search for DNA methylation sites of succinylated genes. The “data.table” (version 1.17.8) and “foreach” packages in R language were used to extract the DNA methylation site data of succinylated genes from the DNA methylation data. The GWAS analysis software plink and the “data.table” and “foreach” packages in R language were used to filter the DNA methylation site data of succinylated genes, with the parameter threshold set the same as the SNP screening criteria in Section 2.2. The “devtools” (version 2.4.5) and “TwoSampleMR” packages in R language were used to conduct ME analysis on the filtered DNA methylation data of succinylated genes and disease data. The “devtools,” “TwoSampleMR,” and “ggplot2” packages in R language were used to conduct mediation analysis with DNA methylation sites as exposure factors, succinylated genes as mediator factors, and diseases as outcomes. The sensitivity analysis was the same as in Section 2.3.

The epigenome-wide association study (EWAS) Data Hub database (https://ngdc.cncb.ac.cn/ewas/datahub/exploration) was used to evaluate the DNA methylation sites. The methylation site results obtained in the previous step were imported into the EWAS Data Hub database to assess whether the methylation sites were located in the key regulatory regions of succinylated genes.

### 2.6. Mediator analysis of succinylation-immune cells-PAH

To explore the relationship between immune cells and succinylation downstream of succinylation, as well as the role of immune cells in mediating the link between succinylation and PAH, the same methodology was applied. The overall impact of succinylation on PAH was assessed through 2 components:

- The direct effect of succinylation on PAH (pathway b in Fig. 1).
- The indirect effect mediated by immune cells (pathways d × e in Fig. 1).

The “data.table” and “foreach” packages in R language were used to filter the genetic data of immune cells, with the parameter threshold set according to the SNP screening criteria in Section 2.2. The “devtools” and “TwoSampleMR” packages in R language were applied to conduct MR analysis on the filtered immune cell genetic data and disease data. The “devtools,” “TwoSampleMR,” and “ggplot2” packages in R language were utilized to perform mediation analysis with succinylated genes as the exposure factor, immune cells as the mediator factor, and diseases as the outcome. The sensitivity analysis was conducted as described in Section 2.3.

### 2.7. Mediator analysis of succinylation-metabolite-PAH

To investigate the relationship between metabolites downstream of succinylation and succinylation itself, as well as the role of metabolites in mediating the connection between succinylation and PAH, the same approach described earlier was employed. The overall impact of succinylation on PAH consists of 2 components:

- The direct effect of succinylation on PAH (pathway b in Fig. 1).
- The indirect effect mediated by metabolites (pathways f × g in Fig. 1).

The “data.table” and “foreach” packages in R language were used to screen the metabolite genetic data, with the parameter threshold set the same as the SNP screening criteria in Section 2.2. The “devtools” and “TwoSampleMR” packages in R language were applied to conduct MR analysis on the screened metabolite genetic data and disease data. The “devtools,” “TwoSampleMR,” and “ggplot2” packages in R language were utilized to perform mediation analysis with succinylation genes as the exposure factor, metabolites as the mediator factor, and diseases as the outcome.

## 3. Results

### 3.1. MR analysis of the relationship between succinylation and PAH

In this study, the IVW method was employed to assess the causal relationship between succinylation-modified genes and PAH. A total of 149 SNPs were selected as instrumental variables (Table S2, Supplemental Digital Content, https://links.lww.com/MD/Q396). The specific effect values of each SNP on PAH can be found in Table S3, Supplemental Digital Content, https://links.lww.com/MD/Q396 and Figures S1a–S10a, Supplemental Digital Content, https://links.lww.com/MD/Q397.

**Figure 2.**
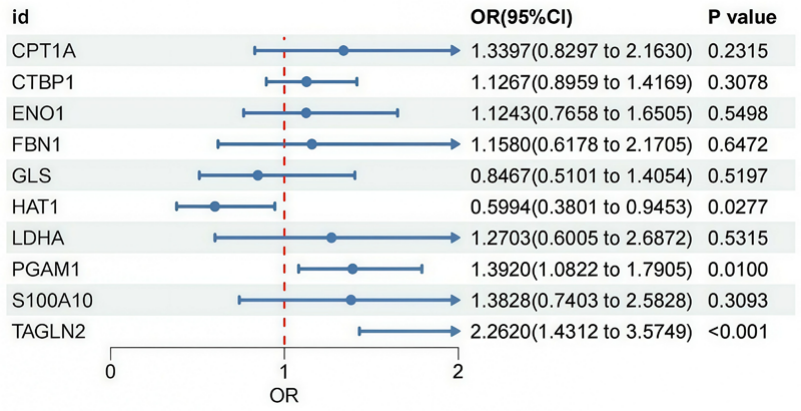
Causality of succinylation genes on PAH risk assessed by the IVW method. CI = confidence interval, IVW = inverse variance weighting, OR = odds ratio, PAH = pulmonary arterial hypertension.

Notably, the *F*-statistics for all selected SNPs were >10, indicating that these instrumental variables possess adequate strength for the analysis. IVW analysis revealed a significant association between HAT1, PGAM1, and TAGLN2 genes and PAH (Fig. 2). Additionally, results from MR-Egger, weighted median, weighted mode, and simple mode methods are provided in Table S4, Supplemental Digital Content, https://links.lww.com/MD/Q396 and Figures S1b–S10b, Supplemental Digital Content, https://links.lww.com/MD/Q397.

The analysis indicated that the HAT1 gene was negatively correlated with PAH risk, suggesting a potential protective role, whereas the S100A10 and TAGLN2 genes were positively correlated with PAH risk, implying that these genes may promote disease development. Figure 2 presents the causal relationship between succinylation-modified genes and PAH risk as assessed by the IVW method.

### 3.2. Bioinformatics analysis and verification of expression levels

**Figure 3.**
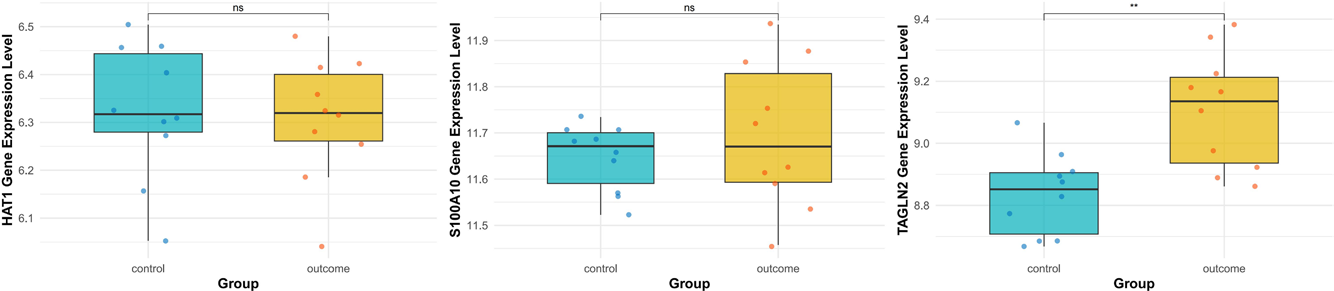
Bioinformatic analysis of HAT1, S100A10, and TAGLN2.

For validation, the GSE131793 dataset from the GEO database was utilized. The limma package in R was applied to perform differential expression analysis on mRNA data from 10 patients with PAH and 10 normal controls. The analysis focused on HAT1, S100A10, and TAGLN2 to examine potential differential expression between patients with PAH and healthy individuals. The results showed no significant difference for HAT1 and S100A10, but TAGLN2 was differentially expressed with statistical significance (*P* < .05), as shown in Figure 3. These results suggest that TAGLN2 may play a role in the pathogenesis of PAH.

### 3.3. Sensitivity analysis

To confirm the reliability of the findings, multiple sensitivity analyses were performed on the TAGLN2 gene. Cochran *Q* test showed a *P* value > .05, indicating no heterogeneity in the MR results. The funnel plot shows basic symmetry on both sides, indicating robust MR results without significant bias (Figure S10c, Supplemental Digital Content, https://links.lww.com/MD/Q397). Since IVW analysis did not detect heterogeneity, a fixed-effect model was used to estimate the causal effect. The intercept of the MR-Egger regression was close to 0 (*P* > .05), indicating the absence of horizontal pleiotropy. The MR-PRESSO test further confirmed no outliers (*P* > .05). Leave-one-out analysis demonstrated that the results remained consistent even after sequentially removing individual SNPs (Figure S10d, Supplemental Digital Content, https://links.lww.com/MD/Q397), further reinforcing the robustness of the MR analysis (Table S5, Supplemental Digital Content, https://links.lww.com/MD/Q396). Overall, all sensitivity analyses support the reliability of the association between TAGLN2 and PAH. The funnel plots and leave-one-out analysis results of other genes are shown in Figures S1c–S9c and Figures S1d–S9d, Supplemental Digital Content, https://links.lww.com/MD/Q397.

### 3.4. Mediator analysis of the relationship between DNA methylation, succinylation, and PAH

In this study, the methylation site data for the TAGLN2 gene was obtained from the NGDC database, and additional relevant data was sourced from the GoDMC database (Table S6, Supplemental Digital Content, https://links.lww.com/MD/Q396). R language was employed to screen for methylation sites using the following criteria: *P*-value < 5e−8; clump_kb = 10,000; clump_r^2^ = 0.1; clump_p = 1. Thus, 8 methylation sites were selected as instrumental variables for subsequent MR analysis (Table S7, Supplemental Digital Content, https://links.lww.com/MD/Q396). The IVW method was applied to analyze the effect of these methylation sites on PAH. The analysis revealed that 4 sites (cg13892570, cg27379915, cg16107628, cg22339338) were significantly associated with PAH (Table S8, Supplemental Digital Content, https://links.lww.com/MD/Q396). Further mediation analysis was conducted, using the 4 methylation sites as exposure variables, the TAGLN2 gene as the mediator, and PAH as the outcome (Table S9, Supplemental Digital Content, https://links.lww.com/MD/Q396). The results indicated that cg13892570 and cg16107628 exerted an indirect inhibitory effect on PAH by regulating TAGLN2 gene expression. The mediation effect of TAGLN2 accounted for 86.46% and 97.65%, respectively (Table 2), suggesting a negative correlation between these 2 loci and TAGLN2 gene expression.

**Table 2:** Mediator analysis results of DNA methylation sites, succinylation, and PAH risk.

ID	Path A	Path B	Total effect	Indirect effect	Direct effect	Adjust the ratio
				a × b	Path C	a × b/c
cg13892570	−0.539252103	0.816234962	−0.509081503	−0.44015642	−0.068925083	0.864608943
cg16107628	−0.613004553	0.816234962	−0.51237479	−0.500355749	−0.012019042	0.97654248

PAH = pulmonary arterial hypertension.

When the IDs cg13892570 and cg16107628 were input into the EWAS Data Hub database, the results showed that the genomic coordinates of cg13892570 were chr1:159893182, located at the TAGLN2 locus, and this site was annotated as “Shore”; the genomic coordinates of cg16107628 were chr1:159894061, also located at the TAGLN2 locus, and this site was annotated as “Shore.”

### 3.5. Mediator analysis of succinylation-immune cell-PAH relationship

Immune cell genetic data were retrieved from the GWAS Catalog database (Table S10, Supplemental Digital Content, https://links.lww.com/MD/Q396). Using R language, immune cells were screened under the following conditions: *P*-value < 5e−8; clump_kb = 10,000; clump_r^2^ = 0.1; clump_p = 1. In total, 612 immune cell types were selected as instrumental variables for subsequent analysis (Table S11, Supplemental Digital Content, https://links.lww.com/MD/Q396). MR analysis identified 37 immune cell types significantly associated with PAH (Table S12, Supplemental Digital Content, https://links.lww.com/MD/Q396). Further analysis revealed that TAGLN2 significantly impacted 2 immune cell types (Table S13, Supplemental Digital Content, https://links.lww.com/MD/Q396). Mediation analysis was conducted, using TAGLN2 as exposure variables, immune cells as the mediator, and PAH as the outcome (Table S14, Supplemental Digital Content, https://links.lww.com/MD/Q396). The results indicated that TAGLN2 indirectly promoted PAH by regulating HLA DR^+^ monocytes, with the mediating effect accounting for 6.15% of the total effect (Table 3).

**Table 3:** Mediator analysis results of succinylation, immune cell, and PAH risk.

ID	Path D	Path E	Total effect	Indirect effect	Direct effect	Adjust the ratio
				d × e	Path B	d × e/b
HLA DR^+^ monocyte	0.224645429	0.223294316	0.816234962	0.050162047	0.766072915	0.061455402

PAH = pulmonary arterial hypertension.

### 3.6. Mediator analysis of succinylation-metabolite-PAH relationship

Genetic data of metabolites were obtained from the GWAS Catalog database (Table S15, Supplemental Digital Content, https://links.lww.com/MD/Q396). Metabolites were screened using R language under the following conditions: *P*-value < 5e−8; clump_kb = 10,000; clump_r^2^ = 0.1; clump_p = 1. A total of 978 metabolites were selected as instrumental variables for subsequent MR analysis. MR analysis identified 57 metabolites significantly associated with PAH (Table S16, Supplemental Digital Content, https://links.lww.com/MD/Q396). Further analysis revealed that TAGLN2 significantly regulated 7 metabolites (Table S17, Supplemental Digital Content, https://links.lww.com/MD/Q396).

Mediation analysis was conducted, using TAGLN2 as exposure variables, metabolites as the mediator, and PAH as the outcome (Table S18, Supplemental Digital Content, https://links.lww.com/MD/Q396). The results indicated that TAGLN2 indirectly promoted PAH progression by regulating cysteinylglycine disulfide and eicosenedioate, with the mediating effects accounting for 3.15% and 5.21% of the total effect, respectively (Table 4).

**Table 4:** Mediator analysis results of succinylation, metabolite, and PAH risk.

ID	Path f	Path g	Total effect	Indirect effect	Direct effect	Adjust the ratio
				f × g	Path b	f × g/b
Cysteinylglycine disulfide	0.089136242	0.288835646	0.816234962	0.025745724	0.790489238	0.03154205
Eicosenedioate	0.091694467	0.463510933	0.816234962	0.042501388	0.773733574	0.052070041

PAH = pulmonary arterial hypertension.

## 4. Discussion

This study, through MR and mediation analysis, elucidated the complex mechanism by which DNA methylation sites (cg13892570 and cg16107628) influence the levels of HLA DR^+^ monocytes, cysteinylglycine disulfide, and eicosenedioate by regulating the expression of the succinylation-related gene TAGLN2, ultimately modulating the risk of PAH. The findings not only provide novel evidence for the epigenetic regulation of PAH but also highlight the potential role of succinylation modifications in the disease’s onset and progression.

### 4.1. Succinylation modification of TAGLN2

Our analysis indicates that the succinylation-related gene TAGLN2 may promote the occurrence and development of PAH, with an odds ratio value of 2.2620 (95% confidence interval: 1.4312–3.5749, *P* < .001). TAGLN2 (Transgelin-2), an actin-binding protein first identified by Stanier et al in 1998, is located on human chromosome 1q21-q25. Its primary function is to regulate the actin cytoskeleton by binding to actin filaments, which plays a critical role in cytoskeletal remodeling, as well as in the contraction and migration of vascular smooth muscle cells.^[19]^

TAGLN2 is highly expressed in smooth muscle and epithelial cells and is also found in non-smooth muscle cells such as endothelial cells, bone marrow cells, and pancreatic cells.^[20–23]^ Moreover, it is widely expressed across various tissues, including the lungs, ovaries, bladder, colon, spleen, and pancreas.^[21,24,25]^ TAGLN2 is abnormally expressed in several cardiovascular and pulmonary diseases,^[26–28]^ but its specific role in PAH remains underexplored. Our study is the first to confirm the causal involvement of TAGLN2 in PAH via MR and further suggests that it may be regulated by succinylation modification.

Succinylation, an emerging PTM, can influence protein function, stability, and interactions. The N-terminal CH domain of TAGLN2 is an important structural basis for promoting G-actin polymerization and stabilizing F-actin, with lysine at position 40 located within this domain.^[29]^ Therefore, succinylation of TAGLN2 K40 (TAGLN2 K40succ) may affect the stability of the actin cytoskeleton.

Zhang et al^[30]^ compared glioma endothelial cells (GEC) and normal endothelial cells through mass spectrometry analysis and found that the level of TAGLN2 K40succ in GEC was significantly increased (15.36 times). Subsequently, western blot and immunohistochemistry were used to verify the high expression of TAGLN2 K40succ in GEC and glioma tissues. In addition, succinylation-mimicking and desuccinylation mutants were constructed, and functional experiments (proliferation, migration, and angiogenesis analysis) confirmed the pro-glioma angiogenesis and pro-tumor growth effects of TAGLN2 K40succ. Based on our research findings, TAGLN2 K40succ may have a promoting effect on the development of PAH. Future studies, including mass spectrometry analysis or succinylated proteomics, can further verify this hypothesis.

### 4.2. DNA methylation inhibits PAH through TAGLN2: the key role of epigenetic regulation

Our analysis demonstrated that cg13892570 and cg16107628 significantly reduced PAH risk by downregulating TAGLN2 expression, with TAGLN2 mediating the majority of the effect (86.46% and 97.65%, respectively). This suggests that TAGLN2 may play a significant role in PAH development and that DNA methylation exerts a protective effect by suppressing its expression.

The 2 methylation sites, cg13892570 and cg16107628, identified in our study are biologically significant regulatory factors of TAGLN2. Both of these sites are located within the CpG island shore of TAGLN2 as annotated in the EWAS Data Hub database. This site is annotated as “Shore,” indicating that it is adjacent to a CpG-rich promoter region. The Shore is the region where DNA methylation changes most frequently and intensely.^[31]^ As these 2 methylation sites are located in the Shore region, their methylation status is likely to directly regulate the binding ability of transcription factors or chromatin modification complexes to DNA, prevent the binding of activators, and lead to the silencing of the TAGLN2 gene.

### 4.3. TAGLN2 promotes PAH through immune and metabolic pathways: a bidirectional regulatory network

Although the downstream effects of TAGLN2 are primarily suppressed by DNA methylation, our mediation analysis revealed that TAGLN2 itself can promote PAH progression through HLA DR^+^ monocytes (mediated by 6.15%) and eicosenedioate (mediated by 5.21%).

HLA-DR^+^ monocytes are functionally significant immune cells characterized by high expression of HLA-DR on their surface, indicating potent antigen-presenting capacity and immune regulatory function. These cells are crucial markers of immune activation and can secrete pro-inflammatory cytokines (such as TNF-α, IL-6, IL-1β) that drive vascular inflammation and pulmonary vascular remodeling.^[32,33]^ Our results suggest that TAGLN2 may enhance its pro-PAH effect by modulating the polarization or activation of monocytes. This aligns with recent studies indicating that monocyte/macrophage infiltration plays a pivotal role in pulmonary vasculopathy in patients with PAH,^[34,35]^ providing causal evidence for this mechanism. Future research could investigate whether TAGLN2 influences the metabolic reprogramming of immune cells (such as glycolysis or fatty acid oxidation) through succinylation modifications, thereby regulating their pro-inflammatory phenotype.

Additionally, TAGLN2 promotes PAH by modulating cysteinylglycine disulfide (mediated by 3.15%) and eicosenedioate (mediated by 5.21%), suggesting that metabolic imbalance is a significant contributor to PAH pathogenesis.

Cysteinylglycine disulfide, a metabolite of glutathione (GSH), may accumulate in response to enhanced oxidative stress, leading to endothelial dysfunction and smooth muscle cell proliferation.^[36–38]^ TAGLN2 may indirectly elevate cysteinylglycine disulfide levels by inhibiting GSH synthesis (e.g., via GCLC inhibition).

Eicosenedioate, a metabolite of omega-6 polyunsaturated fatty acids, may contribute to PAH by influencing lipid signaling or oxidative stress. Our data suggest that TAGLN2 may promote eicosanoid accumulation by regulating lipid metabolism enzymes, such as desaturases or cyclooxygenases, thus exacerbating vascular dysfunction. This finding aligns with recent studies highlighting the role of lipid metabolism disorders in PAH,^[32,39,40]^ suggesting that targeting the lipid metabolism pathway could represent a promising strategy for PAH treatment.

### 4.4. Innovativeness and limitations of the study

This study is the first to integrate DNA methylation, succinylation modification, immune regulation, and lipid metabolism into the pathogenesis of PAH. It is also the first to analyze the PAH mechanism by combining the epigenetic-protein modification-immune/metabolic network, revealing the multilevel regulatory role of TAGLN2. A novel cascade hypothesis, “DNA methylation-TAGLN2 succinylation-downstream effector molecules,” is proposed, offering new insights for the precise classification of PAH. Several innovative conclusions are drawn: TAGLN2 may play a significant role in PAH, with its expression suppressed by DNA methylation, while its function may be regulated by succinylation modifications.

TAGLN2 exerts a pro-PAH effect via immune (HLA DR^+^ monocytes) and metabolic (cysteinylglycine disulfide, eicosenedioate) pathways, highlighting its potential pleiotropic roles in various pathological stages. Future research could focus on: experimentally verifying the succinylation modification of TAGLN2 (e.g., identifying succinylation sites via (liquid chromatography–tandem mass spectrometry); investigating how TAGLN2 regulates the polarization of HLA DR^+^ monocytes (e.g., through single-cell RNA sequencing or metabolic flux analysis); and evaluating the therapeutic potential of targeting TAGLN2 or its downstream metabolites (e.g., using succinylation inhibitors or omega-6 fatty acid antagonists).

While this study provides causal evidence for the epigenetic-succinylation-immune/metabolic-PAH pathway, several limitations remain: the data are primarily derived from European populations, and validation in diverse ethnic groups is necessary. Mediation analysis cannot fully eliminate confounding factors, highlighting the need for experimental validation of the molecular mechanisms. Additionally, the detection of succinylation modifications requires further experimental support.

## Acknowledgments

We thank Bullet Edits Limited for the linguistic editing and proofreading of the manuscript.

## Author contributions

**Conceptualization:** Yu-Shuo Pan.

**Data curation:** Yu-Shuo Pan.

**Formal analysis:** Yu-Shuo Pan.

**Funding acquisition:** Ni-Ni Qu.

**Investigation:** Guo-Rui Xu, Yi-Bing Qin.

**Methodology:** Guo-Rui Xu, Yi-Bing Qin.

**Project administration:** Guo-Rui Xu, Ni-Ni Qu, Tie-Fa Guan, Ming-Zhuo Cao.

**Resources:** Guo-Rui Xu, Ni-Ni Qu.

**Software:** Guo-Rui Xu, Tie-Fa Guan.

**Supervision:** Ni-Ni Qu, Tie-Fa Guan.

**Validation:** Ni-Ni Qu, Yi-Bing Qin, Tie-Fa Guan, Hong-Wei Bai.

**Visualization:** Yi-Bing Qin, Tie-Fa Guan, Hong-Wei Bai.

**Writing – original draft:** Yu-Shuo Pan, Guo-Rui Xu.

**Writing – review & editing:** Yu-Shuo Pan, Ni-Ni Qu, Ming-Zhuo Cao.

## Supplementary Material




