# Supplementary material for: Exploration of upstream and downstream mechanisms of the TAGLN2 gene in pulmonary arterial hypertension
Source: Medicine (Baltimore). 2025 Oct 17;104(42):e45295. doi: 10.1097/MD.0000000000045295 (PMC12537173; doi:10.1097/MD.0000000000045295)

Supplementary Figure S1: Forest plot (a), scatter plot(b), funnel plot (c) and sensitivity analysis (d) of SNPs associated with CPT1A on PAH.

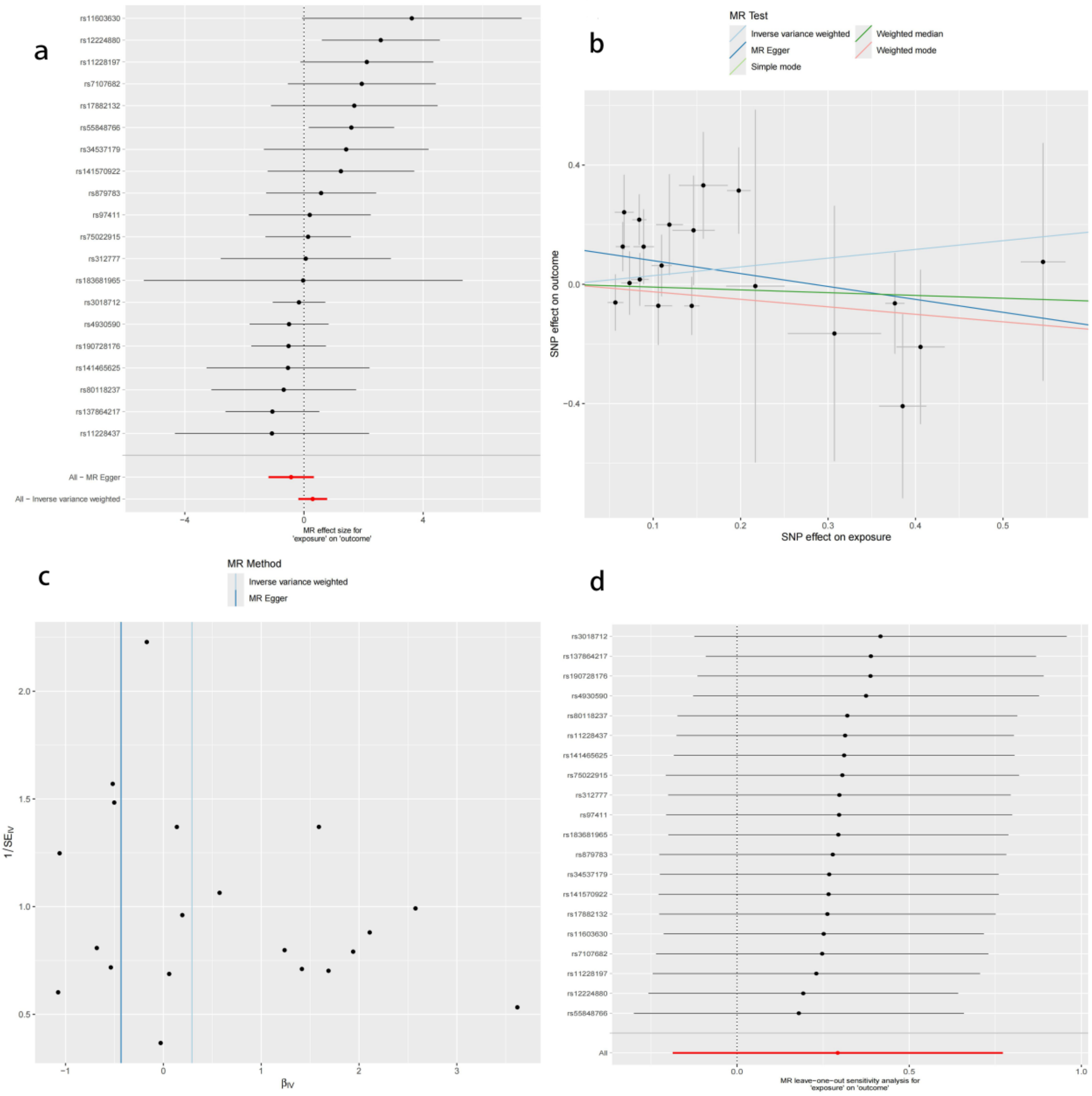

Supplementary Figure S2: Forest plot (a), scatter plot(b), funnel plot (c) and sensitivity analysis (d) of SNPs associated with CTBP1 on PAH.

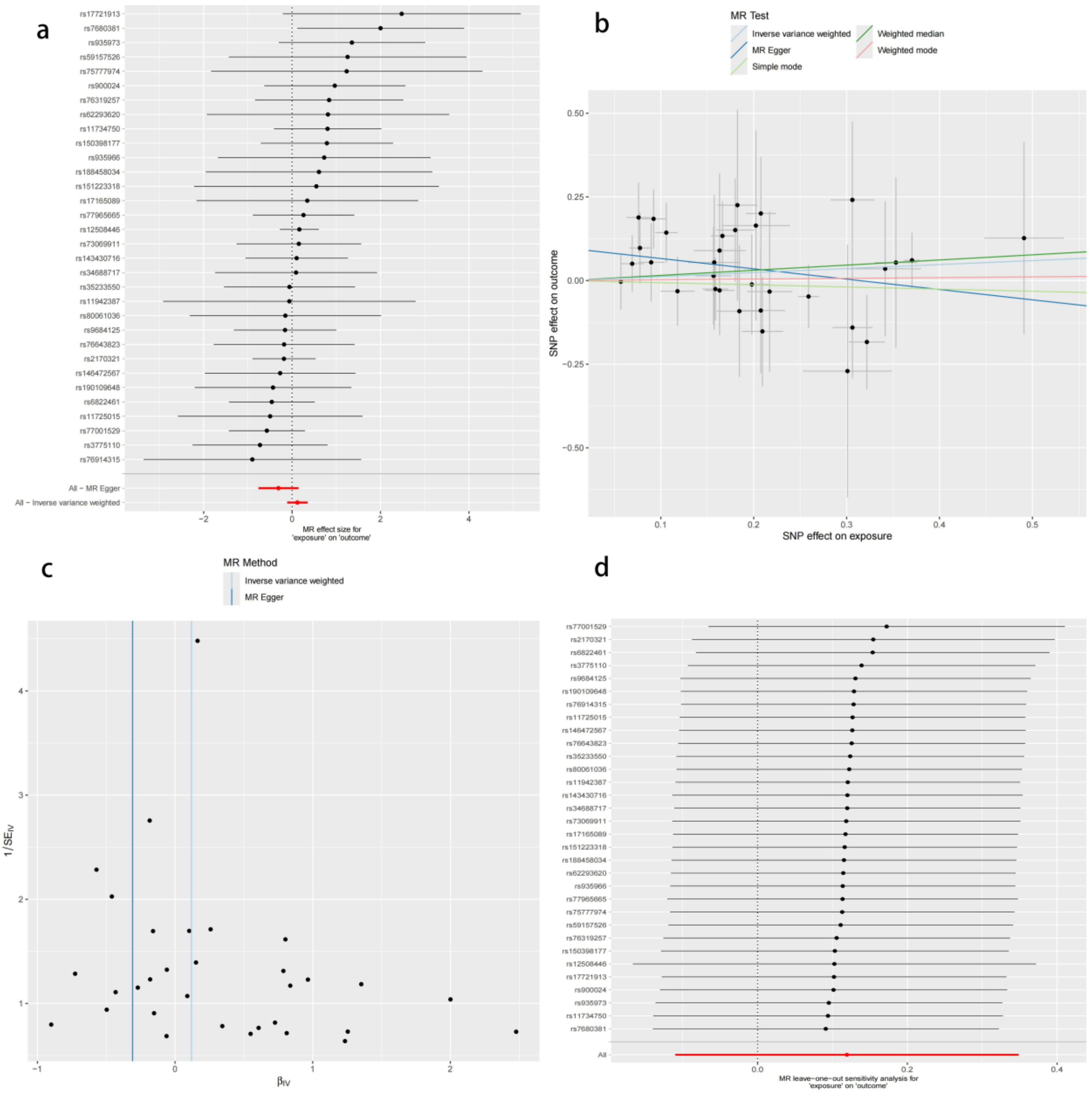

Supplementary Figure S3: Forest plot (a), scatter plot(b), funnel plot (c) and sensitivity analysis (d) of SNPs associated with ENO1 on PAH.

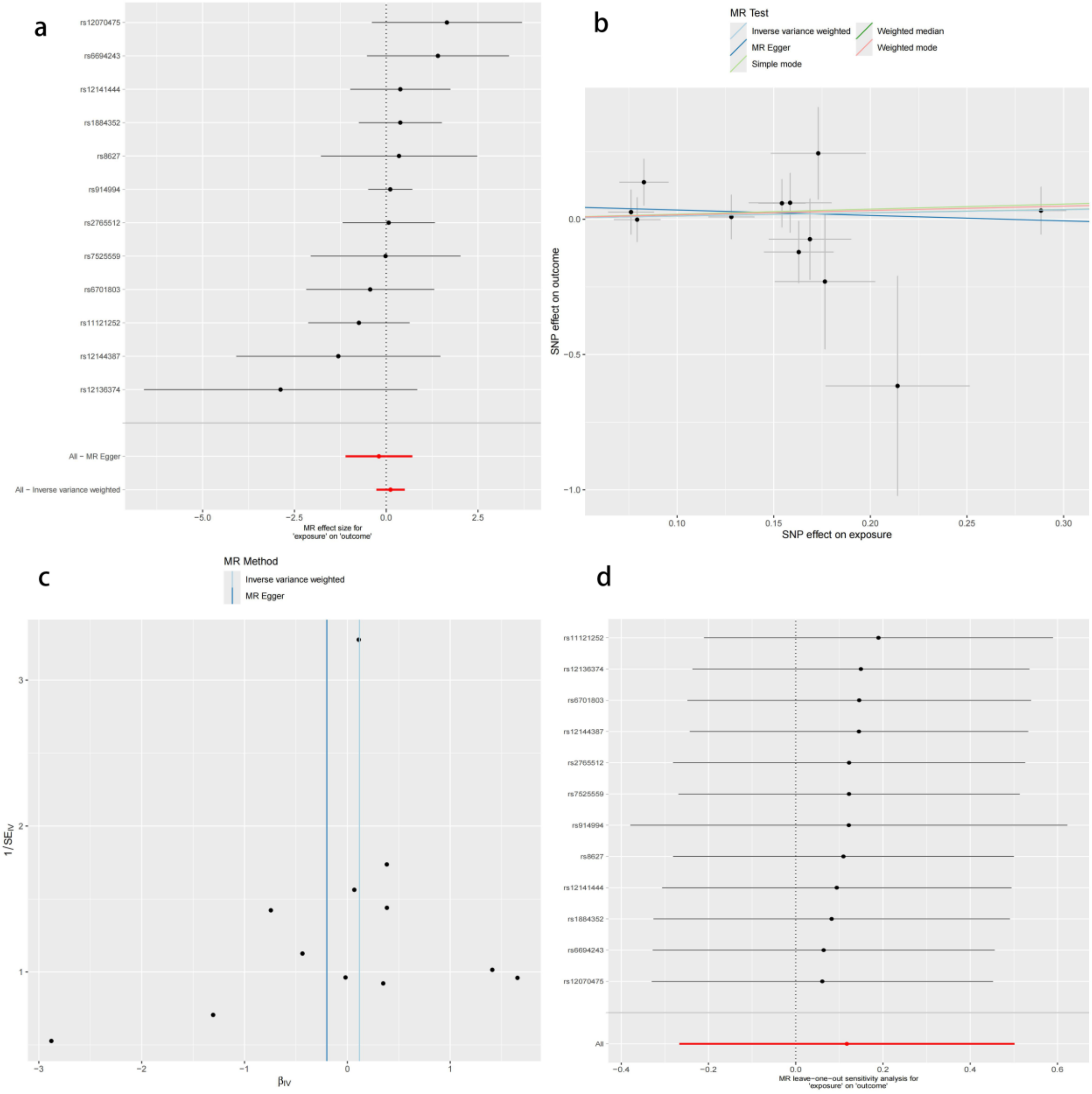

Supplementary Figure S4: Forest plot (a), scatter plot(b), funnel plot (c) and sensitivity analysis (d) of SNPs associated with FBN1 on PAH.

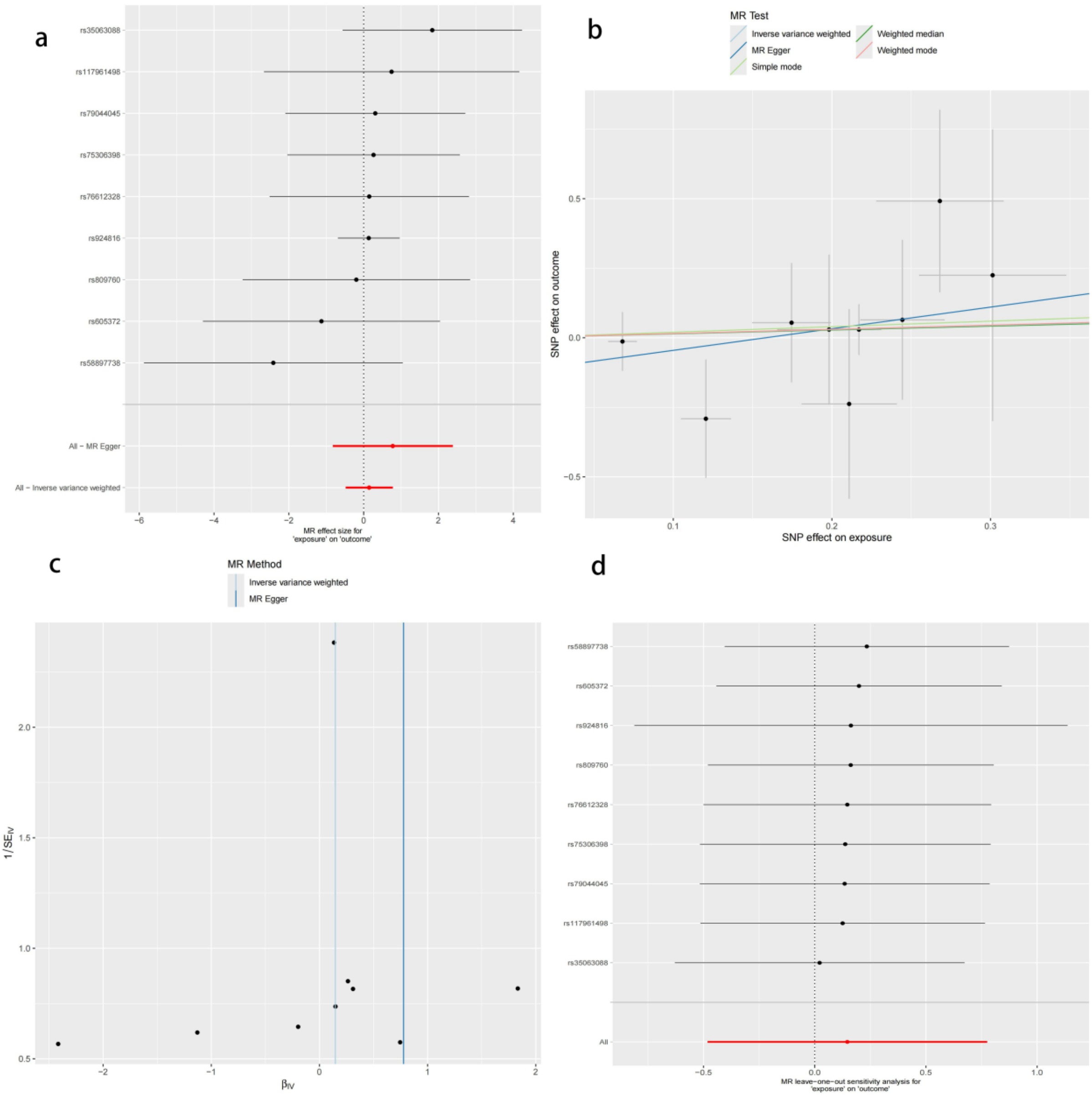

Supplementary Figure S5: Forest plot (a), scatter plot(b), funnel plot (c) and sensitivity analysis (d) of SNPs associated with GLS on PAH.

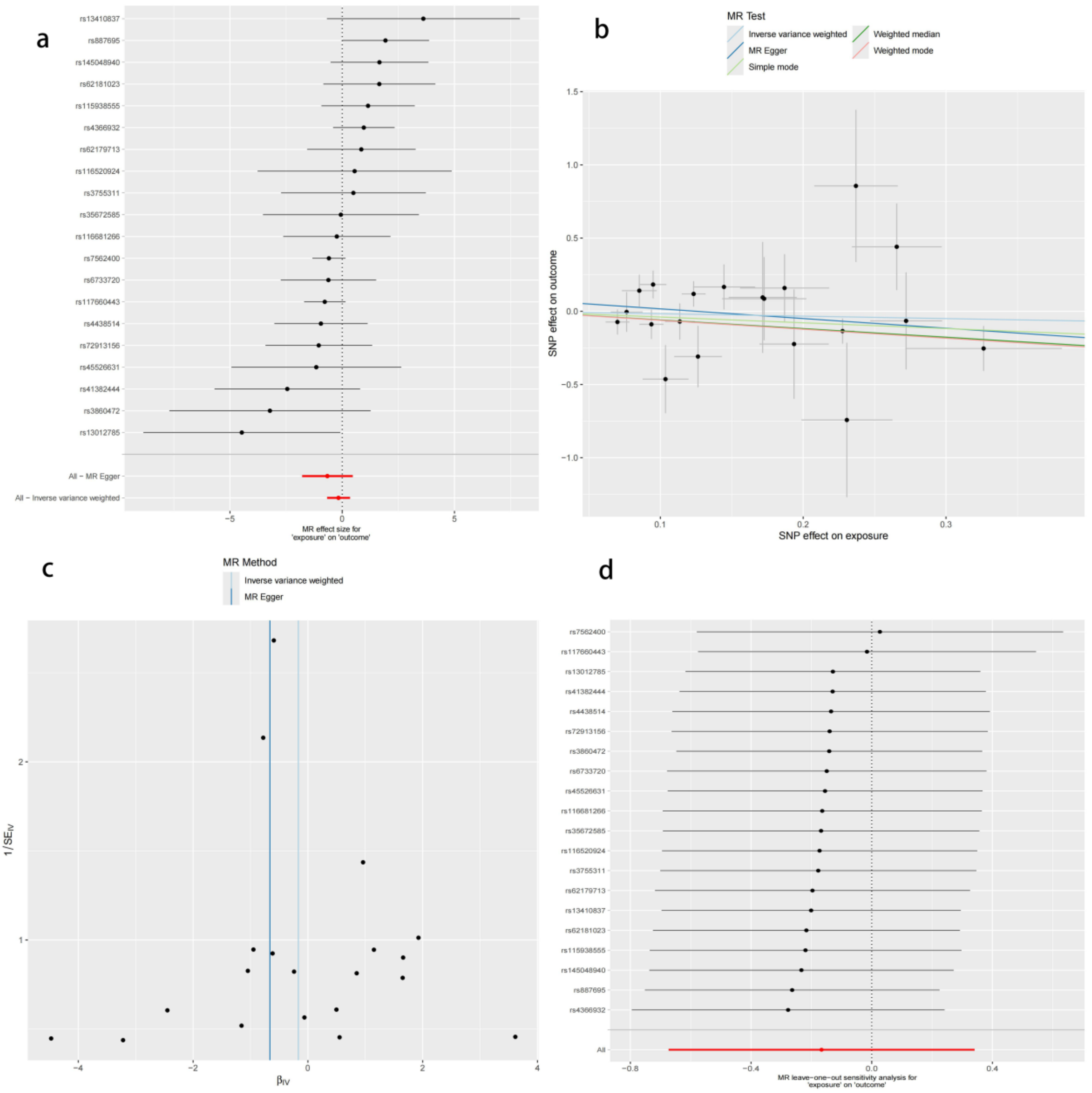

Supplementary Figure S6: Forest plot (a), scatter plot(b), funnel plot (c) and sensitivity analysis (d) of SNPs associated with HAT1 on PAH.

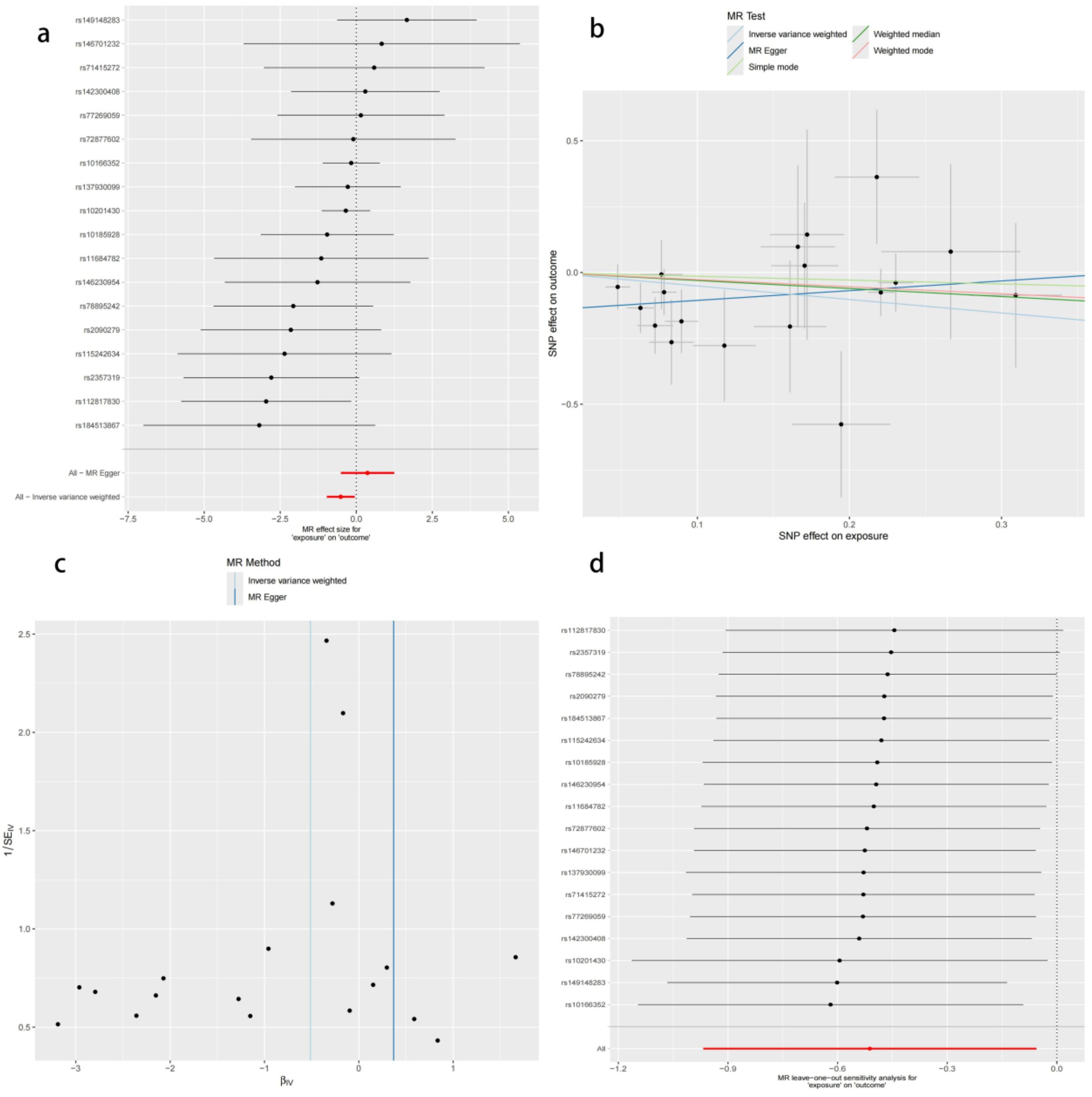

Supplementary Figure S7: Forest plot (a), scatter plot(b), funnel plot (c) and sensitivity analysis (d) of SNPs associated with LDHA on PAH.

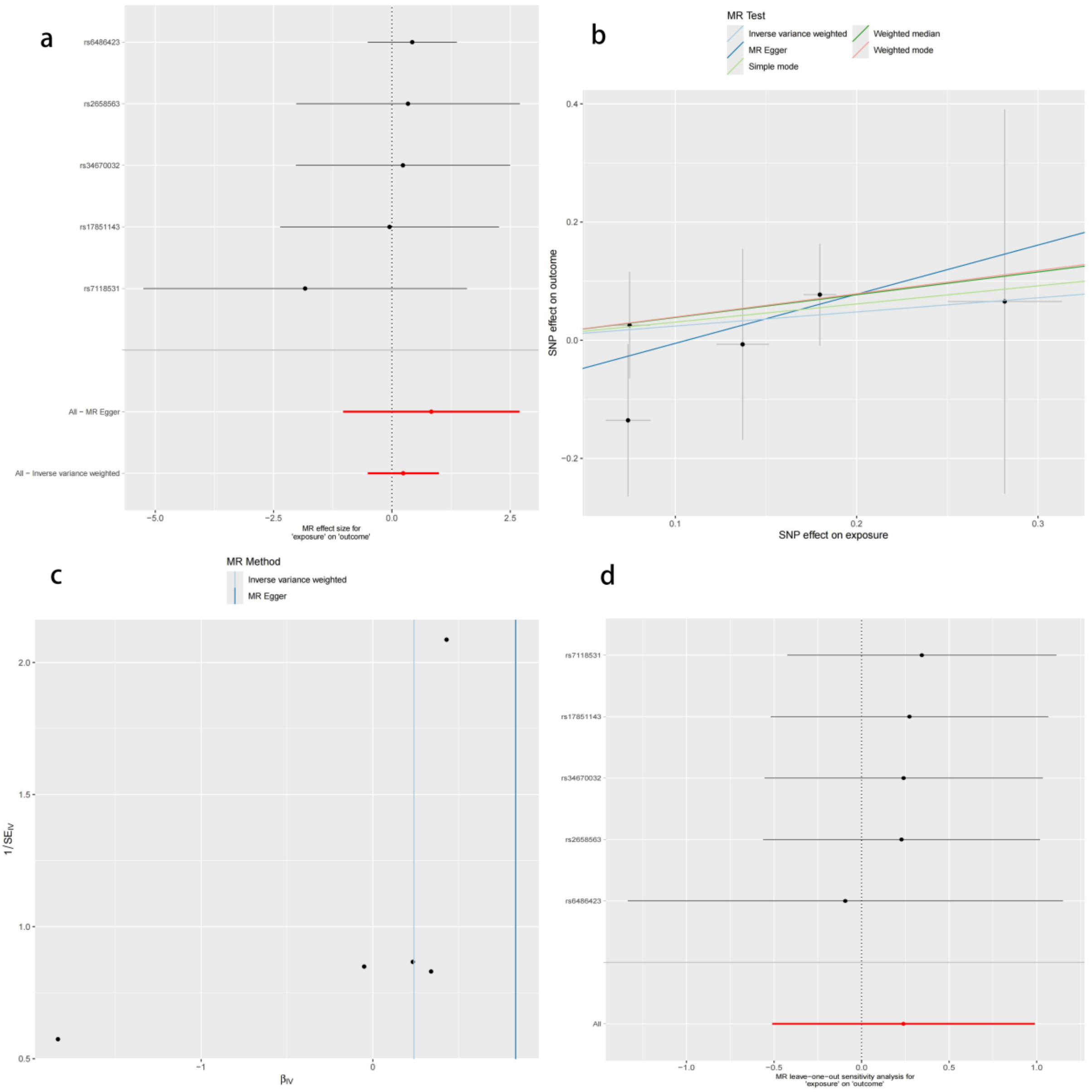

Supplementary Figure S8: Forest plot (a), scatter plot(b), funnel plot (c) and sensitivity analysis (d) of SNPs associated with PGAM1 on PAH.

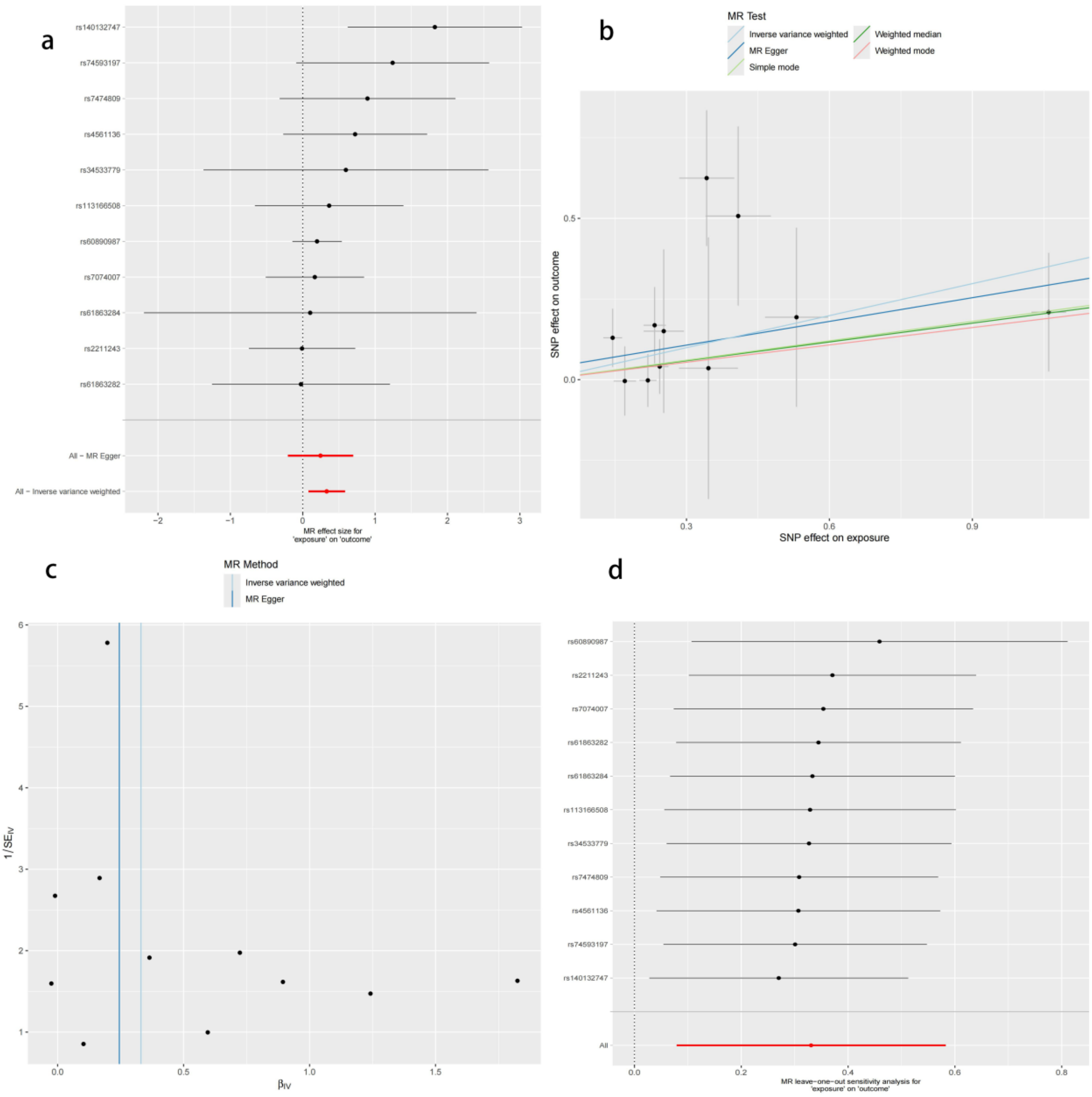

Supplementary Figure S9: Forest plot (a), scatter plot(b), funnel plot (c) and sensitivity analysis (d) of SNPs associated with S100A10 on PAH.

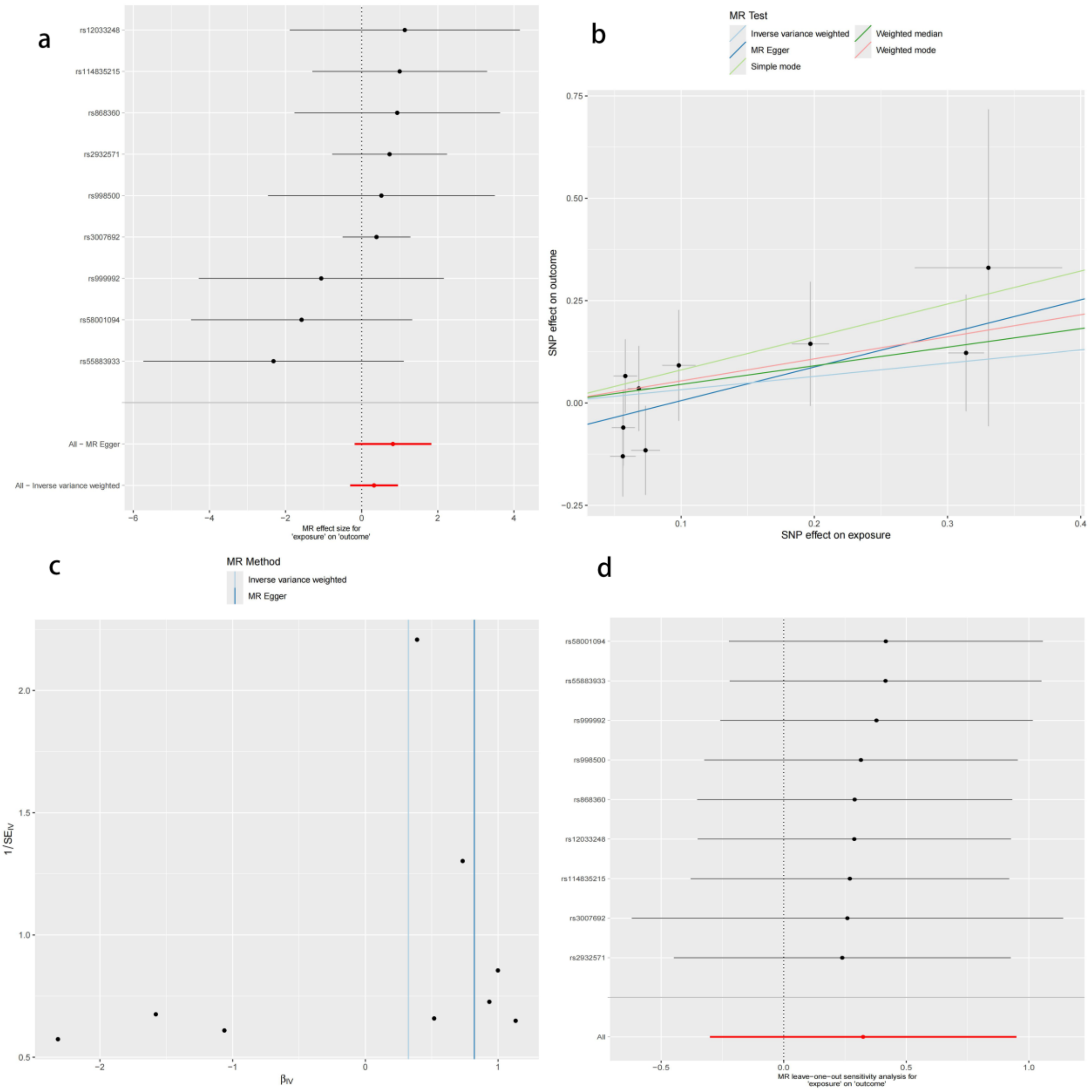

Supplementary Figure S10: Forest plot (a), scatter plot(b), funnel plot (c) and sensitivity analysis (d) of SNPs associated with TAGLN2 on PAH.

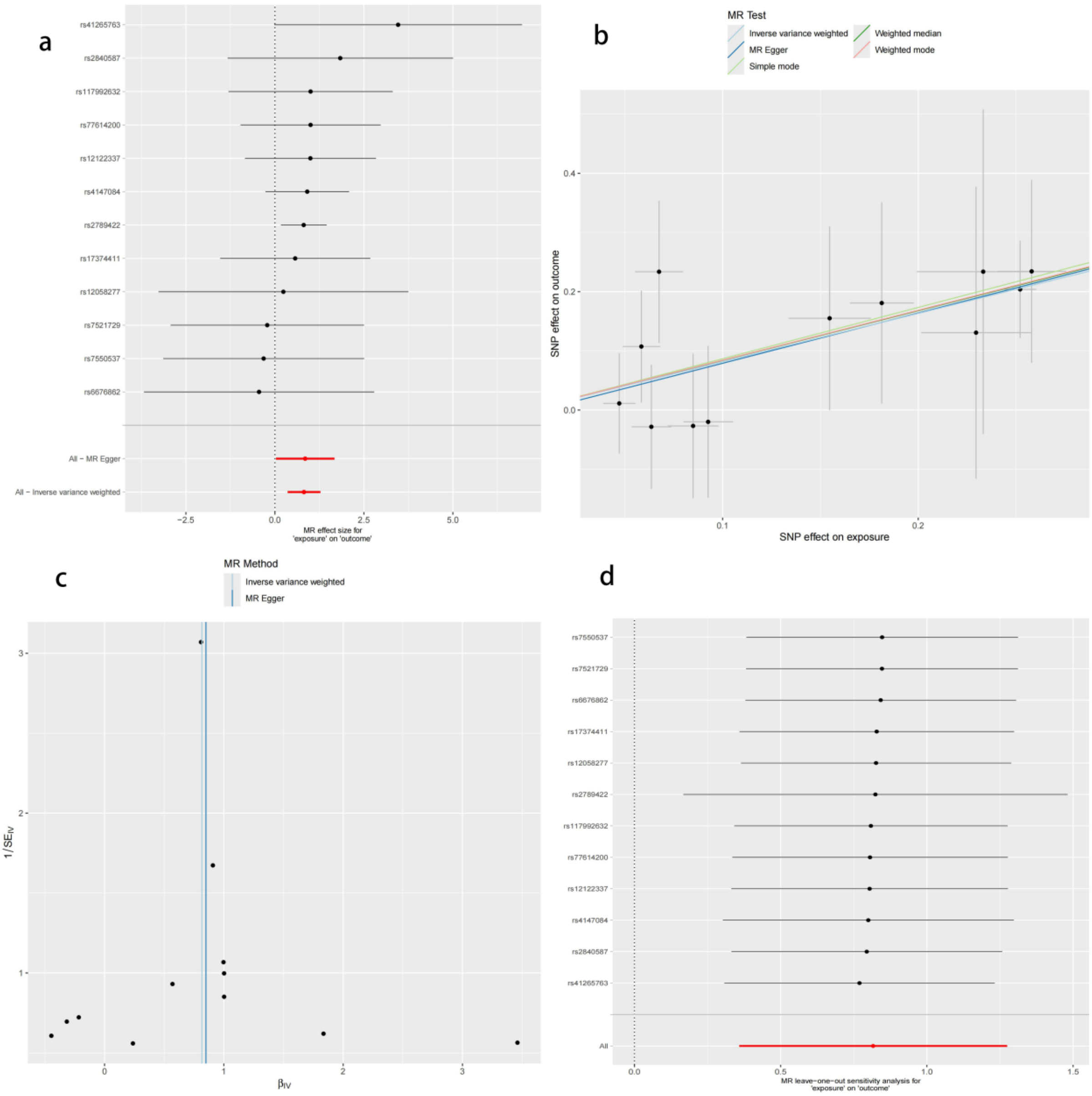

Supplement: Supplementary file 2 [file medi-104-e45295-s002.pdf]
